# Supplementary material for: A Likelihood Approach for Real-Time Calibration of Stochastic Compartmental Epidemic Models
Source: PLoS Comput Biol. 2017 Jan 17;13(1):e1005257. doi: 10.1371/journal.pcbi.1005257 (PMC5240920; doi:10.1371/journal.pcbi.1005257)
Supplement: S1 File — (TAR.GZ) [file pcbi.1005257.s014.tar.gz › HSPH_Online-SI-Revision/MSS10/n10-severe/n10-severe_NEW_table-8.pdf]

1	R0	{1.68952, 11.8402, 1.}	{1.37773, 4.39614, 0.27}	{{1.24751, 2.5364, 0.}, {1.53501, 4.06661, 0.}}	{22.6366, 72.4504, 107.}	inf	217.	94.9696	{27.4936, 139.29}	62.3014	0.482017	newly	155.	226.68	{86., 353.}	64.5677	0.226462	Reff	1.60166	1.32141	{1.19587, 1.47213}	21.508	ar	6872.	4477.22	{3313., 5937.}	38.1147
2	R0	{1.71739, 9.62661, 0.1}	{1.16211, 3.24681, 0.28}	{{1.06738, 2.19254, 0.}, {1.25474, 3.26445, 1.}}	{34.2358, 74.8792, 356.}	inf	89.	42.6578	{6.74253, 67.2183}	67.8863	0.544247	newly	86.	107.28	{19., 195.}	60.8372	0.270848	Reff	1.66844	1.13344	{1.04051, 1.22695}	33.8077	ar	6874.	2100.51	{265., 3895.}	71.1709
3	R0	{1.48936, 6.23975, 0.1}	{1.33133, 4.31628, 0.29}	{{1.22544, 3.07157, 0.}, {1.44485, 5.7404, 1.}}	{16.6239, 46.4467, 354.}	inf	75.	53.1462	{16.1603, 84.0687}	51.8009	0.330162	newly	102.	111.98	{40., 182.}	46.549	0.212414	Reff	1.45406	1.30172	{1.19704, 1.41779}	16.2686	ar	5666.	4100.06	{2915., 5444.}	32.94
4	R0	{1.62394, 7.51135, 1.}	{1.29831, 3.60835, 0.05}	{{1.21159, 2.5364, 0.}, {1.47488, 4.06661, 0.}}	{20.4852, 51.9614, 95.}	inf	51.	34.3197	{5.7251, 64.2051}	46.0707	0.350528	newly	60.	84.54	{15., 164.}	78.8667	0.322075	Reff	1.59877	1.2802	{1.19071, 1.45094}	20.3507	ar	6417.	3850.36	{291., 5745.}	40.614
5	R0	{1.53632, 8.57652, 1.}	{1.1584, 3.486, 0.56}	{{1.06738, 2.19254, 0.}, {1.28596, 5.77111, 2.}}	{24.6424, 59.851, 92.}	inf	91.	36.0905	{9.21248, 56.9782}	60.8662	0.5142	newly	100.	86.57	{33., 145.}	39.31	0.236216	Reff	1.48962	1.12941	{1.03964, 1.25277}	24.2213	ar	6260.	2339.9	{284., 4187.}	62.6214
6	R0	{1.47166, 5.7434, 0.1}	{2.18822, 16.3504, 0.07}	{{2.08951, 15.9744, 0.}, {2.24732, 17.1868, 0.}}	{48.7235, 187.163, 158.}	inf	45.	101.498	{72.0663, 127.095}	127.273	0.349161	newly	63.	58.48	{40., 70.}	21.5556	0.0975933	Reff	1.45297	2.14784	{2.05321, 2.20756}	47.8571	ar	5650.	8352.67	{8053., 8618.}	48.043
7	R0	{1.48179, 8.87369, 0.1}	{1.64629, 9.6158, 1.29}	{{1.28596, 3.98513, 0.}, {1.81391, 11.799, 2.}}	{18.1614, 41.2887, 1254.}	inf	250.	290.113	{114.806, 406.143}	47.3725	0.215993	newly	229.	253.54	{200., 313.}	18.4541	0.0765789	Reff	1.37169	1.51466	{1.20153, 1.66264}	16.7403	ar	5233.	6335.22	{4002., 7486.}	33.2406
8	R0	{1.53669, 6.47807, 0.1}	{2.29073, 13.0802, 0.37}	{{1.93539, 9.63533, 0.}, {2.64334, 17.2462, 1.}}	{49.069, 101.914, 398.}	inf	77.	155.184	{97.9627, 220.256}	102.334	0.288511	newly	107.	113.84	{82., 146.}	18.4112	0.0767706	Reff	1.50611	2.22607	{1.88875, 2.55762}	47.8024	ar	5934.	8496.85	{7748., 9111.}	43.1892
9	R0	{1.57227, 6.20989, 0.1}	{1.82171, 6.86613, 0.58}	{{1.24751, 2.5364, 0.}, {2.71225, 15.0558, 2.}}	{30.4861, 59.2228, 620.}	inf	9.	32.9451	{5.05438, 62.0074}	281.245	0.526792	newly	13.	59.14	{12., 97.}	363.538	0.581373	Reff	1.56598	1.80911	{1.24111, 2.68855}	30.2384	ar	6136.	6209.07	{3175., 9092.}	35.1811
10	R0	{1.54936, 6.50938, 0.1}	{1.88628, 6.23714, 0.31}	{{1.25474, 3.26445, 0.}, {2.65613, 11.0527, 1.}}	{30.032, 36.7205, 360.}	inf	30.	41.2823	{10.9217, 76.9087}	74.6557	0.284932	newly	40.	76.28	{27., 138.}	106.95	0.311986	Reff	1.53789	1.8692	{1.24631, 2.62957}	29.7997	ar	6275.	6728.19	{3603., 9149.}	30.4419
11	R0	{1.42179, 3.18601, 0.1}	{1.29842, 2.22347, 0.}	{{1.06738, 1.89883, 0.}, {1.39162, 2.5364, 0.}}	{9.00453, 30.3286, 100.}	inf	3.	51.8062	{9.28877, 109.856}	1627.99	1.0875	newly	12.	311.79	{33., 795.}	2500.92	1.19033	Reff	1.40601	1.27745	{1.05514, 1.37124}	9.45522	ar	5322.	4029.04	{1713., 5287.}	25.221
12	R0	{1.48035, 6.46183, 0.1}	{2.22796, 10.7836, 0.52}	{{1.47608, 5.83416, 0.}, {2.74056, 14.2212, 2.}}	{52.4622, 74.7439, 544.}	inf	26.	38.9925	{10.4428, 59.9717}	72.8685	0.268876	newly	35.	37.94	{15., 58.}	37.6	0.179566	Reff	1.47147	2.21109	{1.46875, 2.70835}	52.1978	ar	5793.	8114.44	{5904., 9205.}	46.2645
13	R0	{1.75755, 8.53854, 1.}	{1.72749, 10.1714, 0.5}	{{1.22544, 2.5364, 0.}, {2.19504, 17.1709, 1.}}	{19.1032, 60.434, 64.}	inf	33.	33.027	{11.6474, 60.6584}	44.2112	0.232533	newly	37.	34.37	{13., 64.}	43.8108	0.233725	Reff	1.74472	1.71426	{1.21764, 2.17855}	18.9739	ar	6832.	6208.25	{1803., 8477.}	26.6193
14	R0	{1.47355, 4.89451, 0.1}	{2.05259, 10.7396, 0.78}	{{1.22544, 3.57392, 0.}, {2.86095, 18.1089, 2.}}	{45.9032, 132.305, 756.}	inf	20.	57.8828	{13.9766, 105.901}	202.654	0.446217	newly	45.	55.66	{27., 87.}	47.6889	0.18588	Reff	1.4625	2.02772	{1.2178, 2.81822}	45.2669	ar	5710.	7068.15	{3061., 9284.}	45.9799
15	R0	{1.62071, 5.98665, 0.1}	{1.95423, 3.88416, 0.02}	{{1.22544, 2.15811, 0.}, {2.96265, 6.23525, 0.}}	{38.5666, 37.4141, 116.}	inf	11.	10.8032	{3.13096, 22.2837}	56.3401	0.281332	newly	20.	76.57	{6., 219.}	312.45	0.486978	Reff	1.61763	1.95025	{1.22367, 2.9552}	38.5225	ar	6635.	5907.09	{25., 9393.}	44.272
16	R0	{1.67268, 7.7256, 1.}	{1.75468, 6.38097, 0.79}	{{1.24751, 1.89883, 0.}, {2.51417, 16.7164, 3.}}	{23.6334, 77.7267, 121.}	inf	103.	92.1656	{26.1137, 191.609}	53.2452	0.303021	newly	121.	248.4	{93., 489.}	118.992	0.293318	Reff	1.63555	1.71484	{1.22667, 2.43543}	23.0376	ar	6947.	6277.95	{3705., 8973.}	25.4013
17	R0	{1.67805, 11.1417, 1.}	{2.04551, 10.2172, 1.01}	{{1.28596, 3.83841, 0.}, {2.97157, 18.9406, 2.}}	{32.9108, 43.3587, 85.}	inf	109.	84.6041	{24.5556, 148.924}	39.8779	0.25992	newly	87.	85.61	{51., 121.}	28.7931	0.133249	Reff	1.646	2.00912	{1.26952, 2.90032}	32.7404	ar	6625.	7333.75	{4073., 9337.}	28.2667
18	R0	{1.66743, 9.99046, 0.1}	{2.29289, 14.5125, 1.81}	{{1.77826, 9.06807, 1.}, {2.86095, 19.9929, 2.}}	{39.635, 54.275, 1722.}	inf	118.	237.697	{120.454, 351.915}	107.587	0.306339	newly	114.	147.89	{118., 175.}	32.3947	0.121888	Reff	1.61574	2.19055	{1.71619, 2.70873}	37.6436	ar	6899.	8301.83	{7114., 9314.}	24.379
19	R0	{1.70787, 9.85478, 0.1}	{1.77314, 18.8875, 4.89}	{{1.75837, 16.4899, 4.}, {1.79789, 19.6311, 5.}}	{4.27491, 91.6582, 4790.}	inf	143.	212.929	{179.957, 248.656}	49.0975	0.169455	newly	120.	89.02	{72., 106.}	25.85	0.134589	Reff	1.63409	1.68409	{1.66599, 1.71163}	3.51008	ar	6867.	7214.14	{7027., 7414.}	5.50779
20	R0	{1.53809, 10.5848, 1.}	{1.13822, 2.57187, 0.04}	{{1.06738, 2.19254, 0.}, {1.25474, 3.26445, 0.}}	{25.9981, 75.7022, 98.}	inf	127.	35.3962	{8.33965, 62.0309}	72.129	0.663969	newly	110.	112.65	{23., 191.}	48.1909	0.262048	Reff	1.48364	1.10835	{1.03671, 1.22114}	25.2952	ar	6108.	2057.41	{305., 3851.}	66.3161
21	R0	{1.59925, 6.74479, 0.1}	{2.50928, 8.35921, 0.07}	{{1.74092, 4.40955, 0.}, {2.99092, 10.5991, 0.}}	{57.6099, 38.3732, 156.}	inf	35.	66.1787	{24.0904, 100.194}	101.642	0.306892	newly	45.	111.05	{57., 162.}	150.689	0.374386	Reff	1.58662	2.48072	{1.72382, 2.94378}	57.0484	ar	6411.	8632.21	{7131., 9398.}	35.7025
22	R0	{1.38779, 4.40977, 0.1}	{2.23433, 11.9307, 2.25}	{{1.39162, 1.89883, 0.}, {2.64373, 19.7855, 4.}}	{60.9989, 200.425, 2200.}	inf	43.	194.386	{48.837, 281.297}	362.542	0.634887	newly	75.	225.06	{113., 529.}	201.413	0.396534	Reff	1.36101	2.15318	{1.36244, 2.53975}	58.2655	ar	5154.	7932.81	{4913., 9131.}	55.7646
23	R0	{1.53657, 7.50352, 1.}	{1.9858, 10.845, 1.17}	{{1.28596, 3.98513, 0.}, {2.76849, 18.1089, 2.}}	{36.1907, 67.0748, 69.}	inf	100.	113.069	{31.0954, 191.101}	50.9214	0.240502	newly	116.	97.14	{51., 133.}	24.0862	0.137138	Reff	1.50353	1.93772	{1.26339, 2.67154}	35.5428	ar	5928.	7275.11	{4206., 9214.}	35.0137
24	R0	{1.71131, 6.24526, 0.1}	{1.88511, 12.6461, 2.32}	{{1.39162, 1.89883, 0.}, {2.39393, 19.7817, 4.}}	{23.1594, 139.847, 2278.}	inf	12.	47.4004	{10.1484, 78.748}	304.425	0.555925	newly	18.	56.61	{15., 113.}	223.056	0.388412	Reff	1.70104	1.86619	{1.38086, 2.36656}	22.7554	ar	6924.	6915.03	{4895., 8812.}	24.7685
25	R0	{1.4412, 3.07729, 0.1}	{2.11054, 6.42241, 0.15}	{{1.44762, 3.37285, 0.}, {2.9194, 10.1535, 1.}}	{48.4658, 109.952, 224.}	inf	5.	24.3147	{6.56206, 45.4248}	390.946	0.60944	newly	20.	54.17	{18., 114.}	180.25	0.377949	Reff	1.43789	2.10119	{1.44406, 2.90159}	48.1628	ar	5810.	7493.74	{5250., 9344.}	39.9604
26	R0	{1.4159, 4.93858, 0.1}	{2.65438, 9.71338, 0.2}	{{1.9862, 6.28417, 0.}, {2.99092, 10.6768, 1.}}	{87.9685, 98.9531, 266.}	inf	18.	50.1807	{29.256, 72.4597}	180.702	0.425351	newly	30.	69.53	{34., 108.}	134.633	0.343309	Reff	1.40925	2.63306	{1.97191, 2.96057}	87.339	ar	5255.	8917.48	{7972., 9381.}	71.6731
27	R0	{1.57465, 7.05414, 0.1}	{1.91427, 10.8056, 1.12}	{{1.13726, 3.26445, 0.}, {2.70795, 18.9406, 2.}}	{34.6598, 80.5431, 1096.}	inf	15.	8.84303	{2.23907, 16.6118}	48.4444	0.353166	newly	22.	12.78	{2., 29.}	74.2727	0.578751	Reff	1.57134	1.91129	{1.1362, 2.70296}	34.6755	ar	6359.	6392.17	{58., 9192.}	39.9039
28	R0	{1.41422, 4.62402, 0.1}	{2.1666, 11.2385, 0.56}	{{1.40191, 5.7404, 0.}, {2.86095, 18.33, 2.}}	{55.3081, 145.185, 570.}	inf	6.	16.672	{4.12371, 31.4287}	189.671	0.426412	newly	14.	16.73	{4., 36.}	60.3571	0.275043	Reff	1.41153	2.15992	{1.39699, 2.84914}	55.1294	ar	5485.	7743.19	{5014., 9285.}	51.6873
29	R0	{1.41901, 3.63017, 0.1}	{2.02313, 11.7634, 0.79}	{{1.13227, 6.16453, 0.}, {2.91001, 19.0443, 2.}}	{48.6601, 224.584, 778.}	inf	3.	3.87327	{1.45544, 7.26752}	59.5301	0.211625	newly	4.	4.24	{1., 8.}	80.	0.429587	Reff	1.4183	2.02186	{1.1316, 2.9073}	48.6403	ar	5979.	6296.29	{6., 9322.}	51.0221
30	R0	{1.60719, 4.72984, 0.1}	{1.95283, 13.8718, 0.38}	{{1.12882, 8.35044, 0.}, {2.77394, 18.8857, 1.}}	{34.8813, 193.733, 422.}	inf	1.	2.96096	{1.27662, 5.27158}	196.096	0.409095	newly	2.	2.69	{0., 6.}	102.5	0.479966	Reff	1.60687	1.95201	{1.12849, 2.77212}	34.8605	ar	6479.	5593.82	{3., 9203.}	49.1881
31	R0	{1.38175, 6.26468, 0.1}	{1.61775, 3.92204, 0.45}	{{1.39162, 1.89883, 0.}, {2.63407, 13.6136, 3.}}	{17.2738, 77.9977, 520.}	inf	64.	81.6806	{20.1781, 198.864}	72.6737	0.291448	newly	96.	366.36	{109., 692.}	287.688	0.515975	Reff	1.35577	1.58156	{1.36215, 2.54559}	16.8493	ar	5397.	5768.49	{4817., 9097.}	18.8158
32	R0	{1.4947, 8.8847, 1.}	{1.12996, 2.5869, 0.05}	{{1.06738, 2.19254, 0.}, {1.25474, 3.57392, 0.}}	{24.4021, 70.8836, 95.}	inf	208.	66.1626	{19.3217, 109.835}	68.1911	0.60212	newly	186.	190.66	{50., 322.}	46.086	0.256949	Reff	1.38021	1.05934	{0.997687, 1.17559}	23.2474	ar	5407.	2267.91	{892., 3908.}	58.056
33	R0	{1.55891, 8.28978, 1.}	{1.39578, 5.34515, 0.21}	{{1.25474, 3.26445, 0.}, {1.44485, 6.00596, 1.}}	{10.4643, 35.5213, 83.}	inf	149.	108.984	{57.4719, 148.248}	28.8312	0.177625	newly	142.	175.84	{119., 235.}	32.338	0.123336	Reff	1.49203	1.34128	{1.21104, 1.39016}	10.1035	ar	6239.	4975.82	{3725., 5644.}	20.2465
34	R0	{1.60764, 10.4729, 0.1}	{1.36597, 5.91467, 0.21}	{{1.22544, 3.07157, 0.}, {1.38815, 6.71734, 1.}}	{15.8086, 44.4483, 280.}	inf	256.	143.637	{61.853, 197.356}	45.8719	0.303591	newly	188.	196.32	{136., 252.}	20.5	0.0899631	Reff	1.49125	1.28187	{1.1637, 1.30874}	14.7576	ar	6495.	4681.17	{3467., 5378.}	28.6197
35	R0	{1.48527, 5.74485, 1.}	{2.02842, 9.39971, 0.77}	{{1.24751, 3.07157, 0.}, {2.86001, 18.33, 2.}}	{44.6774, 94.7978, 83.}	inf	25.	45.7037	{7.0176, 91.0609}	114.023	0.368666	newly	43.	56.22	{17., 102.}	62.4186	0.251266	Reff	1.47606	2.01003	{1.23881, 2.82507}	44.2573	ar	5811.	6749.53	{1961., 9261.}	44.7457
36	R0	{1.61457, 9.93364, 1.}	{1.28301, 4.31091, 0.34}	{{1.06738, 2.19254, 0.}, {1.28596, 3.98513, 1.}}	{28.2585, 72.4651, 98.}	inf	112.	57.2236	{8.67616, 83.1604}	69.2229	0.550683	newly	110.	114.64	{21., 209.}	54.0364	0.303013	Reff	1.56904	1.25191	{1.04456, 1.26227}	27.5878	ar	6338.	3112.85	{304., 4279.}	56.9415
37	R0	{1.47831, 7.08139, 0.1}	{2.3942, 13.1651, 0.94}	{{1.93419, 8.56054, 0.}, {2.93371, 18.33, 2.}}	{62.8647, 92.004, 916.}	inf	63.	89.6501	{45.9927, 131.603}	53.7441	0.187388	newly	82.	74.44	{44., 100.}	27.6341	0.130764	Reff	1.45954	2.35646	{1.9096, 2.87962}	62.3431	ar	5530.	8519.97	{7681., 9328.}	56.3796
38	R0	{1.73445, 9.8858, 0.1}	{1.30548, 3.89107, 0.24}	{{1.06738, 2.19254, 0.}, {1.43965, 3.98513, 1.}}	{34.793, 72.4434, 314.}	inf	67.	43.5159	{5.78839, 72.8341}	67.3924	0.473744	newly	71.	101.14	{16., 174.}	79.0423	0.314582	Reff	1.70548	1.28471	{1.05193, 1.41587}	34.3888	ar	6842.	3066.04	{435., 5510.}	61.2417
39	R0	{1.66621, 11.4824, 0.1}	{1.5327, 7.24463, 0.58}	{{1.40191, 5.7404, 0.}, {2.01902, 13.4708, 2.}}	{14.5163, 42.7046, 614.}	inf	179.	146.523	{82.4005, 259.381}	37.136	0.191284	newly	134.	174.45	{116., 225.}	36.3955	0.132087	Reff	1.58806	1.46449	{1.34819, 1.89551}	13.8586	ar	6473.	5815.93	{5016., 8040.}	17.6315
40	R0	{1.60694, 9.95741, 1.}	{1.24052, 3.62768, 0.27}	{{1.06738, 2.19254, 0.}, {1.28596, 3.98513, 1.}}	{23.0119, 64.9469, 93.}	inf	141.	50.0575	{17.035, 78.7093}	65.3945	0.525919	newly	132.	120.82	{40., 210.}	41.8182	0.221141	Reff	1.54957	1.20736	{1.04167, 1.25351}	22.2795	ar	6625.	3437.2	{1540., 4257.}	48.3097
41	R0	{1.55128, 4.71911, 0.1}	{2.11723, 9.85723, 0.69}	{{1.27063, 3.98513, 0.}, {2.91001, 18.6082, 2.}}	{44.0029, 115.357, 706.}	inf	6.	5.3143	{1.7062, 10.5671}	52.475	0.279579	newly	17.	9.37	{1., 21.}	75.7059	0.66323	Reff	1.54988	2.11538	{1.27009, 2.90534}	43.9994	ar	6141.	7084.69	{3305., 9326.}	42.1369
42	R0	{1.60063, 6.81118, 0.1}	{1.64469, 13.9635, 0.74}	{{1.33265, 9.41429, 0.}, {1.98261, 16.3049, 2.}}	{11.6705, 109.668, 722.}	inf	35.	30.5914	{12.9188, 44.0161}	29.0931	0.168454	newly	65.	17.84	{9., 27.}	72.5538	0.605848	Reff	1.58799	1.63221	{1.32402, 1.96592}	11.606	ar	6528.	6376.87	{4552., 7837.}	15.4144
43	R0	{1.56986, 5.44652, 0.1}	{2.06769, 10.7311, 0.98}	{{1.27231, 4.98002, 0.}, {2.76849, 17.2836, 2.}}	{39.7742, 104.02, 950.}	inf	8.	10.1447	{2.86598, 19.0701}	67.6026	0.266287	newly	23.	12.01	{2., 18.}	64.8261	0.501586	Reff	1.56751	2.06394	{1.27098, 2.75891}	39.7224	ar	6312.	7043.47	{1138., 9268.}	39.8883
44	R0	{1.47523, 8.07785, 0.1}	{1.62255, 8.97822, 1.07}	{{1.06738, 2.19254, 0.}, {2.1968, 15.4658, 2.}}	{24.2875, 58.9871, 1052.}	inf	101.	88.8465	{15.9226, 182.09}	49.2972	0.333275	newly	100.	85.73	{35., 132.}	32.23	0.19884	Reff	1.43968	1.58321	{1.05101, 2.1205}	23.7822	ar	5692.	5675.94	{1132., 8410.}	38.4491
45	R0	{1.6417, 7.69429, 0.1}	{2.32318, 11.8742, 0.42}	{{1.93419, 6.28417, 0.}, {2.96947, 15.963, 1.}}	{43.2251, 62.2504, 444.}	inf	45.	69.3395	{30.5727, 106.363}	68.6373	0.230199	newly	60.	61.7	{36., 92.}	30.6	0.137054	Reff	1.62545	2.29364	{1.90426, 2.91808}	42.7987	ar	6735.	8416.72	{7679., 9370.}	28.0742
46	R0	{1.38865, 5.83473, 0.1}	{1.31098, 3.27546, 0.}	{{1.06738, 2.19254, 0.}, {1.47608, 4.02511, 0.}}	{9.83268, 43.8627, 100.}	inf	64.	40.4571	{10.9147, 68.1961}	44.4204	0.320535	newly	101.	116.79	{33., 197.}	53.1188	0.246271	Reff	1.35977	1.28671	{1.05135, 1.44702}	9.7501	ar	5233.	3970.52	{1485., 5787.}	29.364
47	R0	{1.45289, 6.87903, 0.1}	{1.2812, 3.56224, 0.09}	{{1.22544, 2.5364, 0.}, {1.44762, 4.00571, 0.}}	{13.5031, 51.9132, 180.}	inf	78.	40.5394	{15.8966, 64.5933}	51.096	0.383446	newly	104.	103.82	{34., 182.}	43.1538	0.236347	Reff	1.42441	1.2607	{1.20306, 1.42621}	13.2097	ar	5379.	3789.32	{1917., 5376.}	31.749
48	R0	{1.69416, 9.4831, 0.1}	{1.71778, 9.2028, 0.23}	{{1.22544, 3.07157, 0.}, {2.2277, 15.555, 1.}}	{20.9068, 47.2867, 292.}	inf	47.	59.4477	{15.2635, 112.86}	62.9713	0.266491	newly	41.	60.1	{31., 88.}	59.0244	0.202131	Reff	1.67264	1.69255	{1.21345, 2.19617}	20.5897	ar	6925.	6264.26	{3342., 8499.}	25.9425
49	R0	{1.47843, 7.16286, 0.1}	{1.37532, 4.40873, 0.52}	{{1.06836, 1.89883, 0.}, {2.22867, 15.6411, 3.}}	{20.1817, 73.8457, 586.}	inf	80.	52.7673	{8.21889, 146.972}	65.303	0.478542	newly	81.	129.7	{26., 271.}	93.5062	0.312903	Reff	1.44664	1.34789	{1.05303, 2.15419}	19.6841	ar	5449.	4056.47	{1641., 8430.}	40.2828
50	R0	{1.68843, 5.72427, 0.1}	{2.00679, 6.40634, 0.4}	{{1.22544, 2.5364, 0.}, {2.86004, 12.7047, 2.}}	{33.9352, 48.4601, 450.}	inf	14.	9.09044	{3.21841, 17.2578}	49.0361	0.333214	newly	26.	27.76	{4., 51.}	79.8462	0.39029	Reff	1.68522	2.00377	{1.22419, 2.85135}	33.9399	ar	6800.	6485.23	{30., 9267.}	38.0381
